# Supplementary material for: Healthcare providers’ perception towards utilization of health information applications and its associated factors in healthcare delivery in health facilities in Cape Coast Metropolis, Ghana
Source: PLoS One. 2024 Feb 1;19(2):e0297388. doi: 10.1371/journal.pone.0297388 (PMC10833587; doi:10.1371/journal.pone.0297388)
Supplement: S1 Table — (PDF) [file pone.0297388.s001.pdf]

Supplementary file 1: Table 6: Descriptive statistics study constructs

Statements	N	Minimum	Maximum	Mean	SD
**Benefits of Information Technology (BoIT) in healthcare delivery**					
Easier to access patient records	632	1	7	5.88	1.48
Easier to find investigation results	632	1	7	5.86	1.38
Helps in preparing hospital report	632	1	7	5.86	1.33
Helps in managing patients	632	1	7	5.6	1.32
Provides speed to accomplish work	632	1	7	5.76	1.38
Saving paperwork	632	1	7	5.81	1.33
Facilitates coordination among departments	632	1	7	5.53	1.52
Improves decisions making process	632	1	7	5.61	1.43
Ensures patients` privacy	632	1	7	5.82	1.28
Reduces medical errors	632	1	7	5.36	1.49
Improves quality of patients' care	632	1	7	5.49	1.25
Decreases workload	632	1	7	5.46	1.59
**Barriers to Information Technology Use (BITU) in healthcare delivery**					
Time-consuming	632	1	7	3.1	1.9
Insufficient number of computers	632	1	7	5.11	1.53
The system is down frequently	632	1	7	5.09	1.52
Low system performance	632	1	7	5.04	1.58
Lack of training for the hospital staff	632	1	7	4.88	1.54
Lack of technical support	632	1	7	4.8	1.6
Incapability of the system	632	1	7	4.73	1.46
Lack of management support	632	1	7	4.79	1.44
**Motives of Information Technology (MoITU) in healthcare delivery**					
Provide new/durable applications	632	1	7	3.1	1.9
Provide training to staff	632	1	7	5.11	1.53
Change the hospital's work procedures	632	1	7	5.09	1.52
Provide technical support	632	1	7	5.04	1.58

1. N – Number of participants; SD - Standard Deviation
2. Source: Field Data (2022)
