# Supplementary material for: Healthcare providers’ perception towards utilization of health information applications and its associated factors in healthcare delivery in health facilities in Cape Coast Metropolis, Ghana
Source: PLoS One. 2024 Feb 1;19(2):e0297388. doi: 10.1371/journal.pone.0297388 (PMC10833587; doi:10.1371/journal.pone.0297388)
Supplement: Attachment — Submitted filename: Response - COMMENTS TO AUTHORS_Plos One -01.08.23.pdf [file pone.0297388.s005.pdf]

31 July 2023

Editor-in-Chief
PLOS ONE

Dear Sir/Madam,

Re: Responses to Reviewers and Editor's Comments

On behalf of my colleagues, I am submitting responses to Reviewers and Editor's comments raised in our article "*Healthcare providers' perception towards utilization of health information applications and its associated factors in healthcare delivery in health facilities in Cape Coast Metropolis, Ghana*".

The following areas of concern raised by the reviewers and Editor with responses (highlighted) as detailed below:

Response to the 1st Reviewer

COMMENTS TO AUTHORS

Let me congratulate authors for taking time to research into such an important phenomenon. I find the following comments useful for enriching the content of the manuscript.

- ✓ *Thank you for your feedback.*

Abstract

The first appearance of the phrase healthcare provider must be in full, and immediately afterwards the acronym HCP follows in a bracket. It is only when this has been done that subsequent sentences can simply use the acronym HCP. I see an acronym HP under methods – this has not been defined in earlier sentences.

- ✓ *Thank you for your feedback. We have provided the full meaning of HCP at the first instance (see line 28)*

"*We conclude that HPs appear to have a positive perception*…" – What precisely do you mean by a positive perception?

Introduction

"*Many IT initiatives have sprung up including the 78 DHIMS and MoTeCH and NHIA initiatives..*"

Be precise about which NHIA initiatives are IT initiatives?

- ✓ *Thank you for your feedback. We revised the manuscript accordingly [see lines 79-92]*

” *Waema [11] and Knuth et al. [12], all bid to improve…”*
Check this sentence again and correct the construction error.

- ✓ *Thank you for your feedback. We revised the manuscript accordingly [see lines 96-98]*

Research Methods

I. The design should rather be stated as **Descriptive Cross-sectional** and not cross-sectional descriptive.
   - ✓ *Thank you for your feedback. We revised the manuscript accordingly [see lines 19 and 124]*

II. You obtained an 88% response rate; what are the implications of this on your statistical power?
   - ✓ *Thank you for your feedback. We revised the manuscript accordingly to read to include the 80% power [see line 159].*
     The implication of the response rate on the power has been included in the manuscript [see lines 211-212]

   Why was a power analysis not performed and your statistical power reported?
   - ✓ *Thank you for your feedback. We have corrected the omission [see line 159]*

III. Check sentence construction errors under heading “selection of facilities”
   - ✓ *Thank you for your feedback. We have corrected the error [see line 165]*

IV. You have indicated the use of a simple random sampling technique – my question is, how were you able to interview staff who were selected through your mobile app generator but were either on annual leave, indisposed, etc.?
   - ✓ *Thank you for your feedback. Staff who were available and selected; and voluntarily consented to participate were included in the study [see line 170]*

V. You mention the use of a Cronbach Alpha test to check for internal reliability among items – My question is, can you state examples of some of the precise variables which were being measured by the items? And also state the precise alpha scores obtained from the Cronbach test? And the interpretation of those scores?]
   - ✓ *Thank you for your feedback. The manuscript has been revised to include some of the precise variables which were being measured by the items and how they were being measured (table 1). [See lines 187-197]*

VI. Can authors explain why they avoided a qualitative study for a phenomenon like this, and what the possible implications of this will be.
   - ✓ *Thank you for your feedback. We were constraint with timelines to submit the project work and so could not include the qualitative aspect.*

Discussion

Can you also discuss the potential limitations of your study; of course, this cannot be a perfect study!

- ✓ *Thank you for your feedback. We have included limitation to the study [see line 453-462]*

Response to the 2nd Reviewer

The study seeks to provide relevant information for policy decisions on the use of health information technology to improve patients' outcomes. However, there are some issues with it that must be addressed by authors:

1. Title: The title could be revise as follows; Healthcare **providers'** perception towards utilization of health information applications in healthcare delivery **in health facilities** in Cape Coast Metropolis, Ghana
   - ✓ *Thank you for your feedback. We have revised the title accordingly [see lines 1-2]*

2. Abstract: this must be improved as there are some grammatical errors that must be corrected. The background to the study should be brief- two or three sentences with the problem well projected will help a lot. Under the study conclusions, the implications of the findings should stand out clearly.
   - ✓ *Thank you for your feedback. We have included the implication of findings [see lines 41, 469-472]*

3. Introduction: Explanation of ICT and Health Information Technology and it/their forms should be projected. Both global level related evidence and evidence from developing countries including Ghana should be extensively used. Authors should also include the rationale for introducing ICT in the health sector. Previously what were the challenges with the use of the paper-based records in the health facilities in the Cape Coast Metropolis? Has the introduction of ICT solved those problems? A description of related studies and their gaps will be required to give a solid foundation for the study.
   - ✓ *Thank you for your feedback. We have revised the manuscript to the challenges of the paper-based patients records management and the piloting of electronic health record etc. [see lines 93-105]*

4. Research methodology: This was not rigorous explained. The strobe approach/checklist should be used to present the findings. Authors should provide detailed information on why the study is a descriptive cross sectional one. A description of the study site will be helpful. A sub heading should be created as **study site** and the rationale for doing the study in the three health facilities well described. Also, lines 110 to 120 should be put together and labelled sampling procedures.
   - ✓ *Thank you for your feedback. We have included the study sites [see 129-149]. These facilities were selected and included in the study because they have deployed patients' electronic health record systems to render healthcare delivery to their clients [see lines 164-166]*

5. Results: Both descriptive and inferential results should be presented. Demographic information of the participants should be accompanied by a table. Data analysis should be properly described. Inferential results should be presented.
   - ✓ *Thank you for your feedback. We have included both descriptive and inferential results [see lines [226-337]*
6. Discussion should be strengthened with related works and implications of the findings extensively described. The study limitations should also be described.
   - ✓ *Thank you for your feedback. We have revised the manuscript accordingly*

7. References: a lot of works have been done around the use of ICT and clients' satisfaction in northern Ghana. Authors should look for such studies to strengthen the quality of the work.

Best wishes

- ✓ *Thank you for your feedback. We have including additional references to strengthen our work*

Thank you for your reconsideration and re-evaluation prior to consideration for publication. My colleagues and I appreciate your time and effort and look forward to hearing from you.

Sincerely,

Richard Okyere Boadu (PhD)
Department of Health Information Management
School of Allied Health Sciences
College of Health and Allied Health Sciences
University of Cape Coast
Cape Coast, Ghana
richard.boadu@ucc.edu.gh
