# Supplementary material for: Healthcare providers’ perception towards utilization of health information applications and its associated factors in healthcare delivery in health facilities in Cape Coast Metropolis, Ghana
Source: PLoS One. 2024 Feb 1;19(2):e0297388. doi: 10.1371/journal.pone.0297388 (PMC10833587; doi:10.1371/journal.pone.0297388)
Supplement: Attachment — Submitted filename: Response - Comments to Authors_Plos One - 06.11.23.pdf [file pone.0297388.s007.pdf]

31 July 2023
Editor-in-Chief
PLOS ONE

Dear Sir/Madam,

**Re: Responses to Reviewers Comments**

On behalf of my colleagues, I am submitting responses to the Reviewers' comments raised in our article "*Healthcare providers' perception towards utilization of health information applications and its associated factors in healthcare delivery in health facilities in Cape Coast Metropolis, Ghana*". The following areas of concern were raised by the reviewers and Editor with responses (highlighted) as detailed below:

INTRODUCTION

It is important to describe briefly challenges associated with using ICT for healthcare delivery.

- ✓ *Thank you for the feedback. We have expanded the introduction section to include some challenges associated with using ICT for healthcare delivery [see lines 73-77].*

METHODS

Data collection tool

Describe how interviews were done. What languages were used during the interview process, where were interviews done and how many interviews were conducted? The authors can rename the heading as DATA COLLECTION TOOLS AND PROCEDURES.

- ✓ *Thank you for the feedback. We have revised the manuscript accordingly [see lines 177, 185-188].*

RESULTS

Line 210, please start with a capital letter; Table 2. Results should be reported in the past. For example, the study revealed that…

- ✓ *Thank you for the feedback. We have revised the manuscript accordingly [see lines 218].*

Discussion

Some typographical errors require redress. Line 360, the word DELIVER should be 'DELIVERY'.

- ✓ *Thank you for the feedback. We have revised the manuscript accordingly [see line 368].*

Thank you for your reconsideration and re-evaluation prior to consideration for publication. My colleagues and I appreciate your time and effort and look forward to hearing from you.

Sincerely,

Richard Okyere Boadu (PhD)
Department of Health Information Management
School of Allied Health Sciences
College of Health and Allied Health Sciences
University of Cape Coast
Cape Coast, Ghana
richard.boadu@ucc.edu.gh
