# Supplementary material for: Healthcare providers’ perception towards utilization of health information applications and its associated factors in healthcare delivery in health facilities in Cape Coast Metropolis, Ghana
Source: PLoS One. 2024 Feb 1;19(2):e0297388. doi: 10.1371/journal.pone.0297388 (PMC10833587; doi:10.1371/journal.pone.0297388)
Supplement: Attachment — Submitted filename: COMMENTS TO AUTHORS_Plos One -28.05.23.docx [file pone.0297388.s003.docx]

**COMMENTS TO AUTHORS**

Let me congratulate authors for taking time to research into such an important phenomenon. I find the following comments useful for enriching the content of the manuscript.

**Abstract**

The first appearance of the phrase healthcare provider must be in full, and immediately afterwards the acronym HCP follows in a bracket. It is only when this has been done that subsequent sentences can simply use the acronym HCP. I see an acronym HP under methods – this has not been defined in earlier sentences.

“*We conclude that HPs appear to have a positive perception*…” – What precisely do you mean by a positive perception?

**Introduction**

“*Many IT initiatives have sprung up including the 78 DHIMS and MoTeCH and NHIA initiatives..”*

Be precise about which NHIA initiatives are IT initiatives?

” *Waema [11] and Knuth et al. [12], all bid to improve…”*

Check this sentence again and correct the construction error.

**Research Methods**

I. The design should rather be stated as **Descriptive Cross-sectional** and not cross-sectional descriptive.
II. You obtained an 88% response rate; what are the implications of this on your statistical power? Why was a power analysis not performed and your statistical power reported?
III. Check sentence construction errors under heading “selection of facilities”
IV. You have indicated the use of a simple random sampling technique – my question is, how were you able to interview staff who were selected through your mobile app generator but were either on annual leave, indisposed, etc.?
V. You mention the use of a Cronbach Alpha test to check for internal reliability among items – My question is, can you state examples of some of the precise variables which were being measured by the items? And also state the precise alpha scores obtained from the Cronbach test? And the interpretation of those scores?
VI. Can authors explain why they avoided a qualitative study for a phenomenon like this, and what the possible implications of this will be.

**Discussion**

Can you also discuss the potential limitations of your study; of course, this cannot be a perfect study!
