# Supplementary material for: Healthcare providers’ perception towards utilization of health information applications and its associated factors in healthcare delivery in health facilities in Cape Coast Metropolis, Ghana
Source: PLoS One. 2024 Feb 1;19(2):e0297388. doi: 10.1371/journal.pone.0297388 (PMC10833587; doi:10.1371/journal.pone.0297388)
Supplement: Attachment — Submitted filename: Feedback for authors plos one 26.06.23.docx [file pone.0297388.s004.docx]

Healthcare Provider’s Perception towards Utilisation of Health Information Applications in Healthcare Delivery in Cape Coast Metropolis, Ghana.

The study seeks to provide relevant information for policy decisions on the use of health information technology to improve patients’ outcomes. However, there are some issues with it that must be addressed by authors:

1. Title: The title could be revise as follows; Healthcare **providers’** perception towards utilization of health information applications in healthcare delivery **in health facilities** in Cape Coast Metropolis, Ghana
2. Abstract: this must be improved as there are some grammatical errors that must be corrected. The background to the study should be brief- two or three sentences with the problem well projected will help a lot. Under the study conclusions, the implications of the findings should stand out clearly.
3. Introduction: Explanation of ICT and Health Information Technology and it/their forms should be projected. Both global level related evidence and evidence from developing countries including Ghana should be extensively used. Authors should also include the rationale for introducing ICT in the health sector. Previously what were the challenges with the use of the paper-based records in the health facilities in the Cape Coast Metropolis? Has the introduction of ICT solved those problems? A description of related studies and their gaps will be required to give a solid foundation for the study.
4. Research methodology: This was not rigorous explained. The strobe approach/checklist should be used to present the findings. Authors should provide detailed information on why the study is a descriptive cross sectional one. A description of the study site will be helpful. A sub heading should be created as **study site** and the rationale for doing the study in the three health facilities well described. Also, lines 110 to 120 should be put together and labelled sampling procedures.
5. Results: Both descriptive and inferential results should be presented. Demographic information of the participants should be accompanied by a table. Data analysis should be properly described. Inferential results should be presented.
6. Discussion should be strengthened with related works and implications of the findings extensively described. The study limitations should also be described.
7. References: a lot of works have been done around the use of ICT and clients’ satisfaction in northern Ghana. Authors should look for such studies to strengthen the quality of the work.

Best wishes
