# Supplementary material for: Healthcare providers’ perception towards utilization of health information applications and its associated factors in healthcare delivery in health facilities in Cape Coast Metropolis, Ghana
Source: PLoS One. 2024 Feb 1;19(2):e0297388. doi: 10.1371/journal.pone.0297388 (PMC10833587; doi:10.1371/journal.pone.0297388)
Supplement: Attachment — Submitted filename: Feedback to authors ICT.docx [file pone.0297388.s006.docx]

**Feedback**

Title of paper: Healthcare providers’ perception towards utilization of health information applications and its associated factors in healthcare delivery in health facilities in Cape Coast Metropolis, Ghana.

Please, the paper looks good now. I have made only a few additional comments for consideration.

INTRODUCTION

It is important to describe briefly some challenges associated with using ICT for healthcare delivery.

METHODS

Data collection tool

Describe how interviews were done. What languages were used during the interview process, where were interviews done and how many interviews were conducted? The authors can rename the heading as DATA COLLECTION TOOLS AND PROCEDURES.

RESULTS

Line 210, please start with a capital letter; Table 2. Results should be reported in the past. For example, the study revealed that…

Discussion

Some typographical errors require redress. Line 360, the word DELIVER should be ‘DELIVERY’.

Best wishes
